# Drug-induced expression of EpCAM contributes to therapy resistance in esophageal adenocarcinoma

**DOI:** 10.1007/s13402-018-0399-z

**Published:** 2018-08-16

**Authors:** Xuan Sun, Robert C. G. Martin, Qianqian Zheng, Russell Farmer, Harshul Pandit, Xuanyi Li, Kevin Jacob, Jian Suo, Yan Li

**Affiliations:** 1 Department of Gastrointestinal Surgery, First Hospital of Jilin University, Changchun, 130021 China; 2 Department of Surgery, Division of Surgical Oncology, University of Louisville, Louisville, KY 40202 USA; 3 Department of Pathophysiology, Basic Medicine College, China Medical University, Shenyang, 110122 China

**Keywords:** EpCAM, Esophageal adenocarcinoma, Barrett’s Esophagus, Adriamycin, Cisplatin, 5-FU, Cancer stem cell

## Abstract

**Background:**

With a less than 5% overall survival rate, esophageal adenocarcinoma (EAC) is one of the leading causes of death in the United States. Epithelial cell adhesion molecule (EpCAM) is a cancer stem cell (CSC) marker that is expressed in various epithelial carcinomas, including EAC. Accumulating evidence indicates that CSC subpopulations can initiate cancer development and, in addition, drive metastasis, recurrence and drug resistance. It has also been reported that EpCAM up-regulation in EAC may lead to an aggressive behavior and, thus, an adverse clinical outcome. Here, we aimed to determine whether treatment with standard chemotherapeutic agents may induce EpCAM expression and, concomitantly, increases in malignant potential and drug resistance in EAC.

**Methods:**

EpCAM expression was assessed in 20 primary human EAC/adjacent normal tissues, as well as in a human EAC-derived cell line (OE-19), in a pre-malignant Barrett’s Esophagus cell line (Bar-T) and in a benign esophageal cell line (HET 1-A), using immunohistochemistry, Western blotting and qRT-PCR, respectively. Drug-induced resistance was investigated in OE-19-derived spheres treated with (a combination of) adriamycin, cisplatin and 5-fluorouracil (ACF) using survival, adhesion and flow cytometric assays, respectively, and compared to drug resistance induced by standard chemotherapeutic agents (CTA). Finally, ACF treatment-surviving cells were evaluated for their tumor forming capacities both in vitro and in vivo using spheroid formation and xenograft assays, respectively.

**Results:**

High EpCAM expression was observed in esophageal cancer tissues and esophageal cancer-derived cell lines, but not in adjacent benign esophageal epithelia and benign esophageal cell lines (HET 1-A and Bar-T). The OE-19 cell spheres were drug resistant and EpCAM expression was significantly induced in the OE-19 cell spheres compared to the non-sphere OE-19 cells. When OE-19 cell spheres were challenged with ACF, the EpCAM mRNA and protein levels were further up-regulated up to 48 h, whereas a decreased EpCAM expression was observed at 72 h. EpCAM down-regulation by RNA interference increased the ACF efficacy to kill OE-19 cells. Increased EpCAM expression coincided with the CSC marker CD90 and was associated with an aggressive growth pattern of OE-19 cell spheres in vivo.

**Conclusions:**

From our data we conclude that an ACF-induced increase in EpCAM expression reflects the selection of a CSC subpopulation that underlies tumor development and drug resistance in EAC.

## Introduction

Esophageal carcinoma ranks among the deadliest malignancies known, with an increasing incidence rate during the past decades [1]. This, coupled with a 5 year overall survival rate of 10 to 15% [1], turns esophageal cancer into an emerging oncologic healthcare problem. Epidemiological studies have shown that over the past few decades the diagnosis has shifted from esophageal squamous cell carcinoma (ESCC) to esophageal adenocarcinoma (EAC) [2]. The low overall survival associated with EAC may be attributed to the fact that patients typically only present once they have developed an advanced stage of the disease. This delay in diagnosis and the lack of effective treatment options for advanced EAC have greatly contributed to the deadliness of the disease. Despite multiple attempts that have been made to combat EAC using various chemotherapeutic agents (CTA) in the past [3–7], the clinical outcome following chemotherapy for advanced disease has remained poor. The most commonly used therapeutic agents include cisplatin/platinum-based drugs, 5-fluorouracil (5-FU) and anthracycline derivatives such as adriamycin. These drugs are often used in combination [7], such as infusional 5-FU with cisplatin or infusional 5-FU with cisplatin bolus dosing, or as a combination of all three in a so-called ACF (Adriamycin-Cisplatin-5-FU) regimen [8].

Epithelial cell adhesion molecule (EpCAM) is a transmembrane glycoprotein that was initially described by Kaprowski et al. [9]. Initial findings revealed an ubiquitous nature of this protein and an over-expression in nearly 100% of colorectal adenocarcinomas. Since these initial discoveries, EpCAM expression has been observed in almost every major epithelial carcinoma [10], including Barrett’s adenocarcinoma and ESCC [11]. The mechanisms through which EpCAM expression may increase the malignant potential of epithelial cells have been postulated to be associated with cell cycle signaling and up-regulation of proto-oncogenic activities [12]. EpCAM contains an extracellular epidermal growth factor-like domain and is known to play a role in the basement membrane adhesion of cells [10]. EpCAM has also been shown to be linked to cellular signaling via the Wnt pathway [13, 14], resulting in an ability to potentiate cancer stem cell (CSC) features. Additional data have shown that EpCAM, through the Wnt pathway, may contribute to resistance to chemotherapy [15]. Previously, we found that EpCAM was up-regulated in hepatocellular carcinoma cells after treatment with chemotherapeutic agents, implying a critical role of EpCAM in cell survival [16]. EpCAM expression has previously been observed in EAC as well [17], but so far its role in this malignancy has remained unclear. A recent study showed that an increase in EpCAM expression after standard CTA treatment was associated with the emergence of residual cells with a mesenchymal stem cell-like phenotype [18], which could explain the increase in drug resistance of these cells. Based on these findings, as well as on its ubiquitous expression in epithelial cancers, EpCAM is currently being evaluated as a potential therapeutic target. The objective of our current study was to determine whether treatment with standard chemotherapeutic agents can induce EpCAM expression, thereby leading to an increased malignant potential and to drug resistance in EAC.

## Materials and methods

### Case selection and immunohistochemical staining

In this study, EAC samples were prospectively collected from patients who had undergone tumor resection. In total 40 samples, consisting of paired benign adjacent tissues and cancerous EAC tissues from 20 patients, were acquired from the James Graham Brown Cancer Center Bio-Repository at the University of Louisville following an approved IRB protocol. The demographics of the 20 study subjects are shown in Table 1. The respective tissues were fixed in 10% buffered formalin for 48 h and processed for paraffin embedding. After embedding, 5 μm sections were mounted onto glass slides and stained with hematoxylin and eosin (H&E) for histopathologic evaluation by two pathologists independently, blinded to the subject’s clinical history. EpCAM protein expression was determined by immunohistochemistry (IHC) using a DAKO EnVisionTM+System Kit (DAKO Corporation, Carpinteria, CA, USA) according to the manufacturer’s instructions. In brief, after deparaffinization and hydration, the slides were washed with TRIS-buffer after which peroxidase blocking was performed for 5 min. After washing, the slides were incubated with a monoclonal mouse anti-EpCAM antibody (1:100; SantaCruz Biotechnology Inc., CA, USA) for 60 min at room temperature. Next, the slides were rinsed and incubated with labeled polymer for 30 min at room temperature, after which the chromogenic substrate diaminobenzidine was added as a visualization reagent. Finally, the slides were counterstained with methyl green. A negative control sample was included in each run.

**Table 1:** Demographics of 20 EAC study subjects

Subjects	20	
Gender (male:female)	18:2	
Mean age (year)	67	
Specimens	Adjacent benign tissues (20)	EAC tissues (20)
EpCAM positive staining specimens	2/20	20/20

### Cell lines and culture conditions

SV40 large T transfected esophageal epithelial HET 1-A cells (ATCC, Manassas, VA, USA) were used as a representative of benign esophageal mucosa. Human hTERT-immortalized nonneoplastic Bar-T cells were used as a representative of Barrett’s Esophagus mucosa. Bar-T cells were a generous gift from R. Souza and S.J. Spechler, Department of Medicine, VA North Texas Health Care System and the University of Texas Southwestern Medical School, Dallas, TX, USA. OE-19 cells (Sigma-Aldrich, St Louis, MO, USA), derived from an adenocarcinoma of a gastric/esophageal junction of a 72 year-old male patient, were used as a representative of esophageal adenocarcinoma. HET 1-A cells were maintained in Bronchial Epithelial Cell medium (BEGM BulletKit, Clonetics Corporation, Walkersville, MD, USA) supplemented with penicillin (100 U/ml), streptomycin (100 μg/ml) and 10% fetal bovine serum (FBS). Bar-T cells were maintained in keratinocyte basal medium 2 (KBM-2; Clonetics Walkersville, MD, USA) and OE-19 cells were maintained in Dulbecco’s Modified Eagle Medium (DMEM) (Invitrogen, Carlsbad, CA, USA), respectively, supplemented with penicillin (100/ml), streptomycin (100 μg/ml) and 10% FBS (Invitrogen, Carlsbad, CA, USA). The cells were cultured in 75 ml flasks (Greiner Bio-One, Monroe, NC, USA) at 37 °C with 5% CO_2_. All cell lines have been authenticated (Bio-Synthesis Inc., Lewisville, TX, USA).

### Rationale for drug dosing

Adriamycin, 5-FU and cisplatin are agents typically chosen for CTA treatment of EAC. The concentrations of these drugs used were determined based on extrapolations from two main factors, i.e., clinically applicable dosing and known pharmacokinetic data [19–21]. The dosage of the drugs was standardized based on an average patient size of average height and the approximate median of the range seen in multiple dosing protocols [22–27]. By multiplying the dose and our standardized size, we were able to determine the number of milligrams administered. This dosage was combined with the volume of distribution for each therapeutic agent to create a theoretical chemotherapeutic concentration available in the total body fluid. This concentration was subsequently divided by 5 to account for the presence of the respective drugs in the extra-cellular fluid alone, as that would be the amount that comes into contact with the tumor cells.

### Cell sphere, drug treatment and survival assays

For generating cell spheres, OE-19 cells were seeded into ultra-low attachment 60 mm dishes (Corning, New York, NY, USA) and grown in serum-free DMEM/F12 medium (Invitrogen, Carlsbad, CA, USA) supplemented with 5 μg/ml insulin (Sigma-Aldrich), 20 ng/ml human recombinant epidermal growth factor (Invitrogen, Carlsbad, CA, USA), 10 ng/ml basic fibroblast growth factor (Invitrogen) and 0.4% bovine serum albumin (Sigma-Aldrich). Once cell spheres were formed, they were exposed to culture media alone or culture media supplemented with chemotherapeutic agents for various periods (6, 12, 24, 48 and 72 h) to determine cell survival. The cell spheres were either exposed to single chemotherapeutic agents or to specific combinations of chemotherapeutic agents (i.e., adriamycin, cisplatin and 5-FU; ACF) to mimic the clinical treatment of EAC patients (see above). Cell survival was assessed using a MTT assays as reported before [28]. In brief, following treatment cell spheres as well as dissociated cells were collected and washed with phosphate buffered saline (PBS). Next, the cells were incubated in 100 μl 1 mg/ml MTT suspended in PBS for 4 h, after which the MTT was removed and 100 μl dimethyl sulfoxide (DMSO) was added and incubated for another 10 min. Finally, the results were expressed as absorption measured using an optical density (OD) plate reader at 570 nm. To correct for background noise, the absorbance of DMSO + MTT in wells without cells was subtracted. The mean OD values from triplicate wells for each treatment were used as the index of cell survival. To confirm the MTT results, cell adhesion assays were performed using 6-well plates pre-coated with 40 μg/ml Collagen I solution in PBS. This is a commonly used assay to test the effect of drug treatment in cancer cells. After the cell spheres were exposed to chemotherapeutic agents for 72 h, 10 mM EDTA in DMEM was used to dissociate them. After complete dissociation, the cells were washed, added to each of the Collagen I-coated wells and incubated at 37 °C for 20 min to allow the cells to adhere to the surface. Next, non-adherent cells were washed off four times in DMEM, after which DMEM supplemented with 10% FBS was added and the cells were incubated at 37 °C for 48 h to allow recovery. Finally, computer-based imaging was applied for cell number counting and image capture using an Olympus 1X51 microscope (Olympus, Pittsburgh, PA).

### Flow cytometry

After drug treatment, OE-19 cells were gently digested with 0.025% trypsin-EDTA (1 mM, Invitrogen) and collected by centrifugation. Next, the cells were washed with cold PBS and re-suspended in fluorescence-activated buffer (PBS containing 1% fetal calf serum), after which 100 μl of the cell suspension was mixed with a FTIC conjugated anti-EpCAM monoclonal antibody (EBA-1, BD Biosciences, CA, USA) and incubated for 30 min at room temperature. After washing, the cells were evaluated using a BD FACS Canto II flow cytometer (BD Biosciences). FlowJo 7.6.1 software was used for data analysis.

### Western blotting

Total protein was extracted on ice using a lysis buffer, after which an equal volume loading buffer (100 mM Tris/HCl (pH 6.8), 4% SDS, 20% glycerin, 10% β-mercaptoethanol and 0.2% bromophenol blue) was added and mixed. Next, the samples were denatured at 95 °C for 5 min, loaded on 10% SDS gels and electrophoresed at 100 V. After membrane transfer, the membranes were probed with a mouse monoclonal anti-EpCAM antibody (BD Biosciences; 1:1000 dilution) or a mouse monoclonal anti-GAPDH antibody (Sigma 1:5000 dilution) at 4 °C overnight. After washing, a second donkey anti-mouse HRP antibody (BD Biosciences; 1:2500 dilution) was added. Finally, antibody-antigen complexes were detected using an ECL Western blot detection kit (Pierce, Thermo Scientific, Rockford, IL). The protein bands were quantified by densitometry analysis.

### RNA interference assay

To define the link between drug treatment and EpCAM expression, a small interfering RNA (siRNA) specific for its mRNA (TACSTD1, SI03019667) and a negative control siRNA (1022076) were designed and synthesized by Qiagen (Qiagen, Valencia, CA, USA). OE-19 cells were seeded at a concentration of 4 × 10^5^ cells per well in a 6-well plate after which transfections were performed using Lipofectamine 2000 transfection reagent (Invitrogen) according to the manufacturer’s instructions. In total 100 pmol/well siRNA was used for each transfection. After a 24-h transfection period, the cells were exposed to drugs and subjected to Western blot and cell adhesion assays.

### Quantitative RT-PCR

After drug treatment, total RNA was extracted using TRIzol reagent (Invitrogen). First-strand complimentary DNA (cDNA) was synthesized from total RNA according to the manufacturer’s protocol for the RNA PCR kit (Promega, Madison, WI, USA). Quantitative PCR was carried out using an ABI7300 real-time PCR system (Applied Biosystems, Carlsbad, CA). The comparative cycle time (Ct) method was used to determine fold differences between samples. EpCAM expression was normalized to β-actin as an endogenous reference (2^−ΔΔCt^). The results were expressed as fold changes in gene expression.

### Immunofluorescence and immunohistochemical staining

OE-19 cells were seeded onto 8-well chamber slides and exposed to ACF, cisplatin or 5-FU for 48 h. Next, the cells were fixed in 100 μl 4% formalin in PBS for 10 min, washed with PBS and incubated with FITC-conjugated monoclonal anti-EpCAM antibody (1:100 dilution; BD Biosciences, CA, USA) and PE-conjugated monoclonal anti-CD90 and anti-CD44 antibodies (1:100 dilution; BD Biosciences, CA, USA) at room temperature for 2 h. After this, the cells were washed again and images were captured using an Olympus IX51-DP72 image-system (Pittsburgh, PA, USA). Immunohistochemical staining was performed on paraffin-embedded tumor tissue sections. Endogenous peroxidase was blocked with 3% hydrogen peroxide and 5% goat serum to prevent non-specific reactions. Next, the tissue sections were incubated with primary anti-EpCAM and proliferating cell nuclear antigen (anti-PCNA, ab29; Abcam, Cambridge, MA, USA) antibodies, after which horseradish peroxidase-conjugated secondary antibodies (1: 300–400 dilutions with PBS) were used to detect the primary antibodies. Finally, the tissue sections were incubated with a peroxidase substrate DAB kit (Vector Laboratories, Inc., Burlingame, CA, USA) to develop a brown color. Counterstaining was performed using hematoxylin or methyl green. Digital images were acquired using an Olympus IX51 microscope equipped with Olympus DP72 digital software (Olympus, Pittsburgh, PA, USA) at 20× magnification.

### Xenograft mouse model

Eight-weeks-old nude BALB/c mice were used for xenografting. The mice were given commercial chow and tap water and maintained at 22 °C on a 12-h light/dark cycle. To establish xenografts, OE-19 cell spheres were pretreated with ACF for 6, 12, 24, 48 or 72 h. After treatment, cell sphere cultures were continued to assess their sphere forming capacity. OE-19 cell spheres after 48 h ACF pretreatment were selected for xenografting and inoculated at 10^6^ cells/mouse into the right flank. In total eight mice were used and all manipulations were performed under sterile conditions. Tumor growth was monitored for 5 weeks and tumor sizes were determined by measuring their length and width with an accuracy of 0.01 mm using a digital caliber. Tumor sizes were calculated using the formula: tumor volume = length × width × width/2. The experimental procedures were approved by the Institutional Animal Care and Use Committee of the University of Louisville, which is certified by the American Association for Accreditation of Laboratory Animal Care.

### Statistics

The data are expressed as mean ± standard deviation (*n* = 3-6). Analysis of variance (ANOVA) and Newman-Keuls’ multiple-comparison tests were used for data analysis. Differences between groups were considered significant when *p* < 0.05.

## Results

### High EpCAM expression in esophageal cancer tissues and esophageal cancer-derived cell lines

**Fig. 1:**
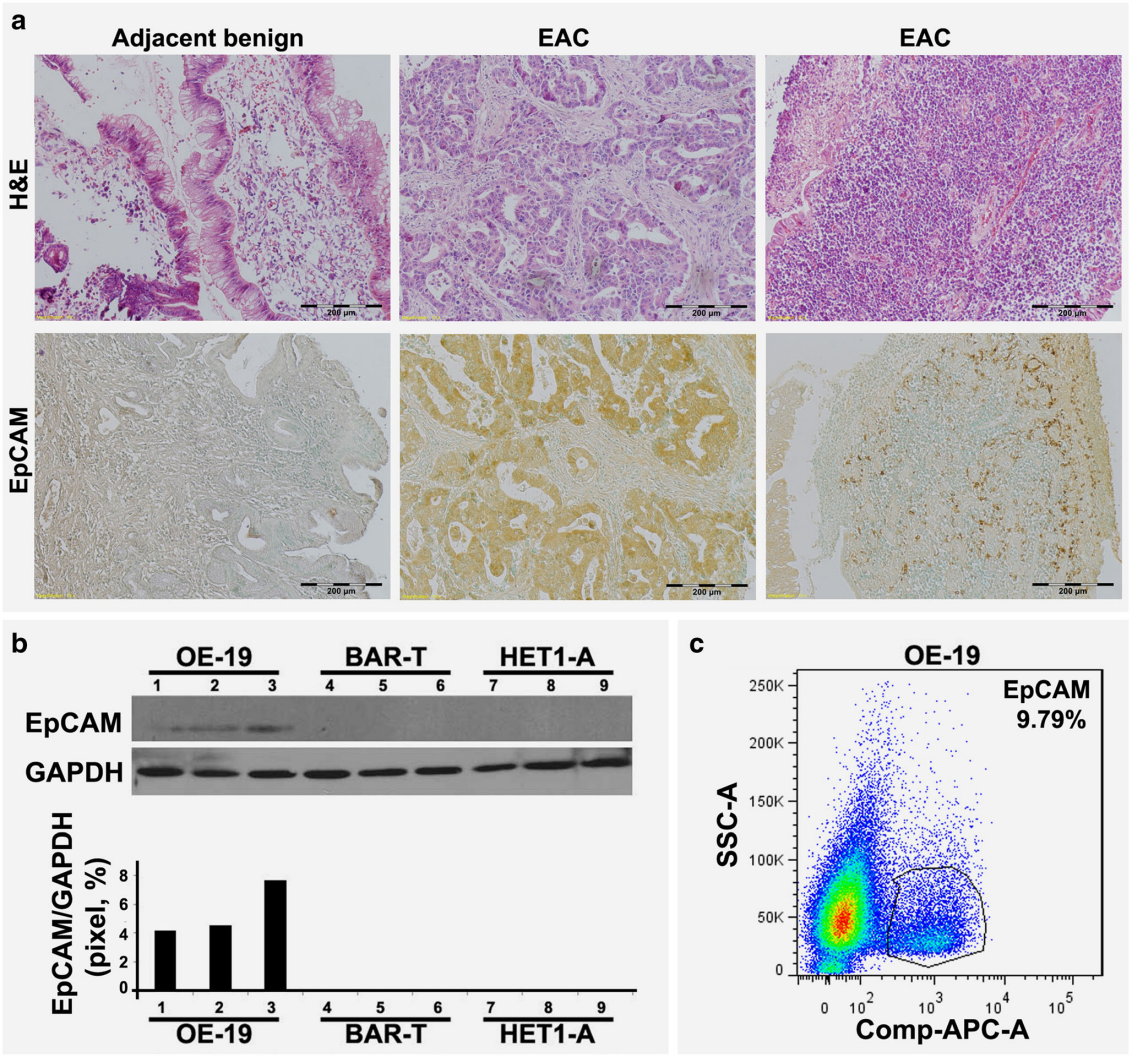
**EpCAM expression in primary human EAC samples and human EAC-derived cells.** (**a**) Representative histological changes of human EAC tissues compared to adjacent benign tissue (upper panels). Immunohistochemical EpCAM staining of human EAC tissue compared to adjacent benign tissue (lower panels). Positive staining is represented as brown color. (**b)** Western blot analysis of EpCAM expression in malignant OE-19 cells, pre-malignant Bar-T cells and benign HET1-A cells. (**c**) Flow cytometric analysis of the proportion of EpCAM positive OE-19 cells

Using immunohistochemistry, we could confirm previous observations that EpCAM is highly expressed in EAC tissues, but not in adjacent benign esophageal epithelia (Fig. 1a). It is widely accepted now that EAC is the end result of a stepwise process of transitions from benign gastroesophageal reflux disease (GERD) to premalignant Barrett’s Esophagus to malignance. Here, the expression of EpCAM was determined by Western blotting in three cell lines, HET 1-A representing benign esophageal epithelium, Bar-T representing pre-malignant Barrett’s metaplasia and OE-19 representing esophageal adenocarcinoma, respectively. We found that the OE-19 cells showed a higher EpCAM expression than the HET 1-A and Bar-T cells (Fig. 1b). The percentage of EpCAM positive OE-19 cells was further evaluated using flow cytometry and, by doing so, we found that the proportion of cells showing positive EpCAM expression was 9.79% (Fig. 1c). Based on these results, we considered this EAC cell line to be suitable for drug challenging experiments to assess the potential role of EpCAM in CSCs contributing to drug resistance with clinical significance.

### Increased cell survival after drug challenge of OE-19 cell spheres

**Fig. 2:**
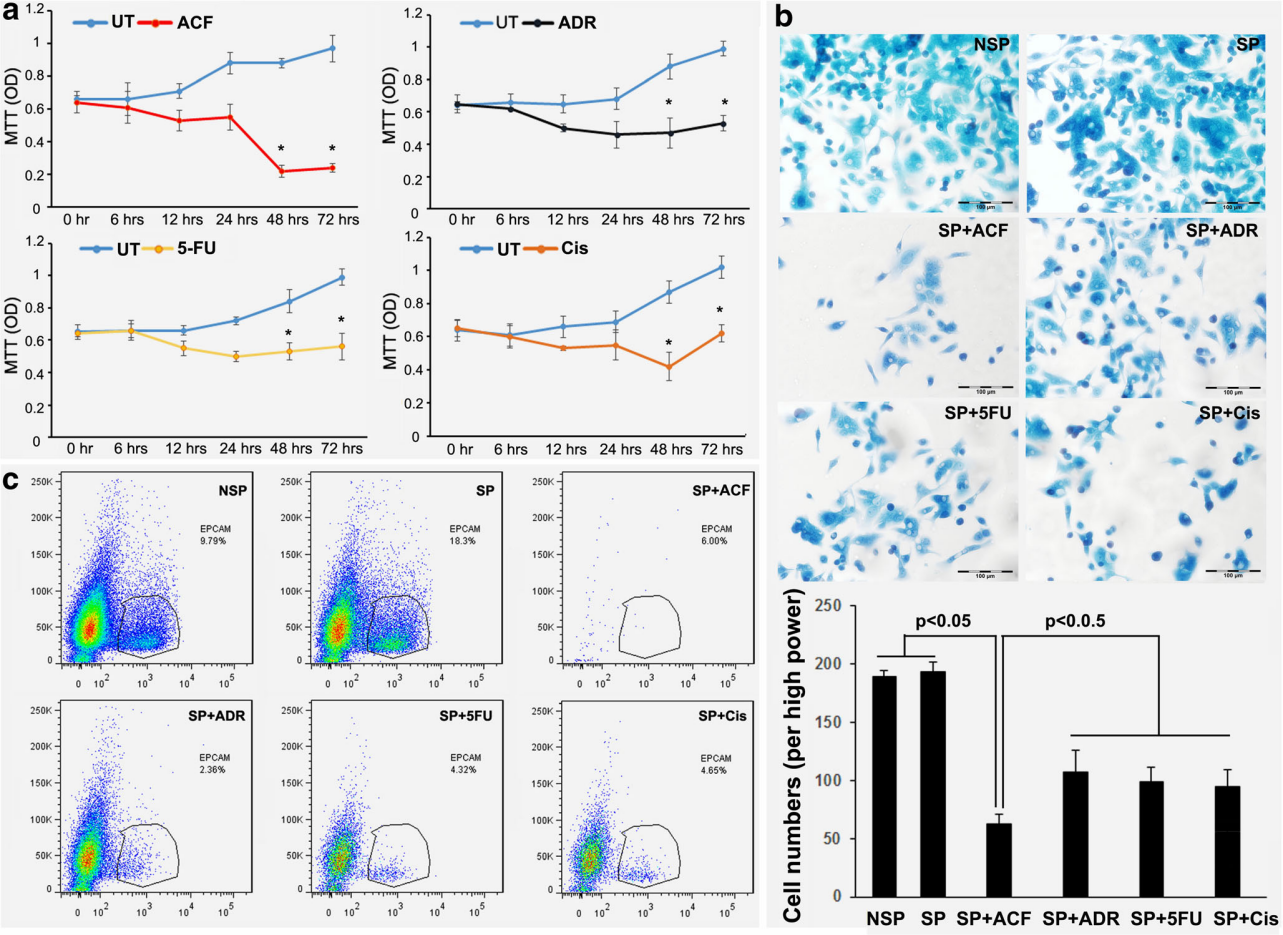
**Cell viability changes and percentage of EpCAM positive cells after drug treatment.** (**a**) MTT assay of spheroid OE-19 cells treated with adriamycin, 5-fluorouracil, cisplatin or its combination (ACF). ADR: Adriamycin; 5-FU: 5-fluorouracil; Cis: cisplatin. (**b**) Cell adhesion assay of spheroid OE-19 cells treated with adriamycin, 5-fluorouracil, cisplatin or its combination (ACF). NSP: non-spheroid OE-19 cells; SP: spheroid OE-19 cells. (**c**) Flow cytometric analysis of the proportion of EpCAM positive cells in OE-19 spheres treated with adriamycin, 5-fluorouracil, cisplatin or its combination (ACF). NSP: non-spheroid OE-19 cells; SP: spheroid OE-19 cells

When patients receive anti-cancer therapies most tumor cells are usually killed, but subpopulations of cancer cells that are resistant to the treatment may remain and give rise to relapse. This phenomenon is thought to be due to the presence of CSCs [29–31]. Therefore, we first performed a sphere formation assay to test the ability of the OE-19 cell line to form putative CSC-derived spheres. We found that OE-19 sphere formation occurred 2–3 days after replacing the culture medium with DMEM/F12 (see materials and methods). To assess whether the OE-19 cell spheres were drug resistant, the cultured spheres were exposed to individual drugs as outlined in Table 2, as well as to combinations of adriamycin, cisplatin and 5-FU (ACF). The OE-19 cell spheres were exposed to each agent during different time periods (0, 6, 12, 24, 48 and 72 h) at concentrations as outlined in the materials and methods section (rationale for drug dosing). Interestingly, we found that most surviving cells started to grow after 6 to 24 h as determined by MTT assay, and continued to do so after 24 h for each individual drug. These abrupt upswings in survival and growth were not mirrored by the combination ACF treatment at 24 h, but a slight upswing was observed after 48 h (Fig. 2a). To confirm these MTT results, cell adhesion assays were performed. A 48 h treatment period was selected based on the MTT results showing that 48 h was the critical time point for drug resistance in the single agent treatments compared to that of ACF. We found that the cell adhesion assay results were consistent with those from the MTT assay (Fig. 2b). In order to subsequently test whether EpCAM positive cells could be responsible for this drug resistance, flow cytometry was performed on the OE-19 cell spheres challenged by both the individual drugs and their combination (ACF) for 48 h. We observed a significant increase in EpCAM positive cells (18.3%) compared to the baseline percentage (9.79%). Consistent with the MTT results, we found that treatment of the OE-19 cell spheres with the individual drugs, as well as with ACF, caused cell death after 48 h. Unexpectedly, we found that the percentage of EpCAM positive cells after ACF treatment was even higher than that after the individual drug treatments, reaching 6%, even though the total amount of surviving cells was less (Fig. 2c). Together, these results indicate that EpCAM expression may be induced by CTA treatment and may be associated with CSC survival and drug resistance.

**Table 2:** Drug concentrations at clinically relevant doses

Drug	Dose	Concentration
Adriamycin	30 mg/m^2^	20.01 μg/ml
5-FU	500 mg/m^2^	28.90 μg/ml
Cisplatin	75 mg/m^2^	0.84 μg/ml

### Increased EpCAM expression after drug challenge of OE-19 cell spheres

**Fig. 3:**
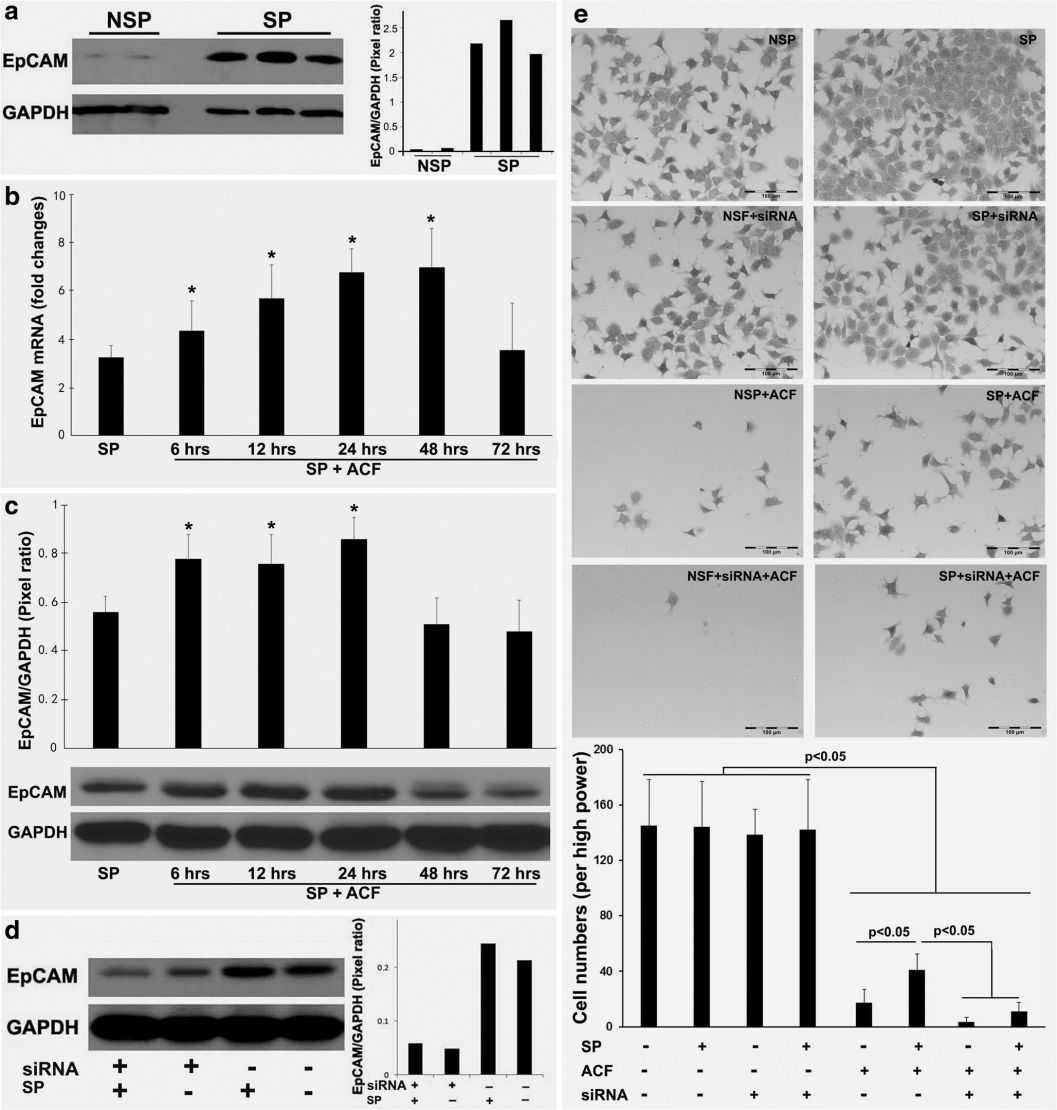
**EpCAM expression in spheroid and non-spheroid EAC cells and its effect on cell viability.** (**a**) Western blot analysis of EpAM expression in non-spheroid and spheroid OE-19 cells. NSP: non-spheroid OE-19 cells; SP: spheroid OE-19 cells. (**b**) qRT-PCR analysis of EpAM expression in OE-19 spheres treated with the drug combination ACF. SP: spheroid OE-19 cells. *p < 0.05 versus SP. (**c**) Western blot analysis of EpAM expression in the OE-19 spheres treated with the drug combination ACF. SP: spheroid OE-19 cells. *p < 0.05 versus SP. (**d**) siRNA-mediated EpCAM downregulation in non-spheroid and spheroid OE-19 cells. SP: spheroid OE-19 cells. ** (e)** Cell adhesion assay of non-spheroid and spheroid OE-19 cells treated with the drug combination ACF for 48 h after EpCAM downregulation. NSP: non-spheroid OE-19 cells; SP: spheroid OE-19 cells

Based on the above results, we next set out to determine the EpCAM expression pattern during sphere formation, as well as its expression pattern related to the cell survival over time after ACF treatment. Firstly, we determined the EpCAM expression levels by Western blotting in OE-19 cells and OE-19 cell spheres over time. We found that EpCAM expression was significantly induced in the OE-19 cell spheres compared to the non-sphere OE-19 cells (Fig. 3a). Next, we challenged the OE-19 cell spheres with ACF at the sensitive concentrations determined previously. Using qRT-PCR analyses, we found that the EpCAM mRNA levels changed over time during ACF treatment. Specifically, we found that EpCAM was up-regulated by ACF from 6 to 48 h, whereas a decreased EpCAM expression was observed at 72 h (Fig. 3b). We also determined the EpCAM protein levels in the OE-19 cell spheres challenged with ACF. Consistent with the mRNA results, we found that the protein levels increased over the time from 6 to 24 h. Decreases in EpCAM protein levels were observed at 48 and 72 h (Fig. 3c). These decreases were found to be associated with decreases in cell viability. Together, these results indicate that changes in EpCAM expression may play a role in the drug resistance of surviving cells after ACF treatment. Since the cell viability and flow cytometry results indicated that a certain percentage of cells survived after ACF exposure after 48 h, the surviving cells may have acquired CSC-like features. Next, a RNA interference assay was performed in OE-19 cells to assess whether EpCAM down-regulation may increase ACF efficacy. We found that RNA interference effectively caused EpCAM mRNA and protein level decreases to about one third compared to the negative (scramble) control (Fig. 3d). A subsequent cell adhesion assay revealed that after 48 h of ACF treatment most non-spheroid OE-19 cells were killed, either with or without RNA interference, whereas a more efficient cell kill was observed in the spheroid OE-19 cells (Fig. 3e).

### Increased EpCAM expression coincides with the CSC marker CD90

**Fig. 4:**
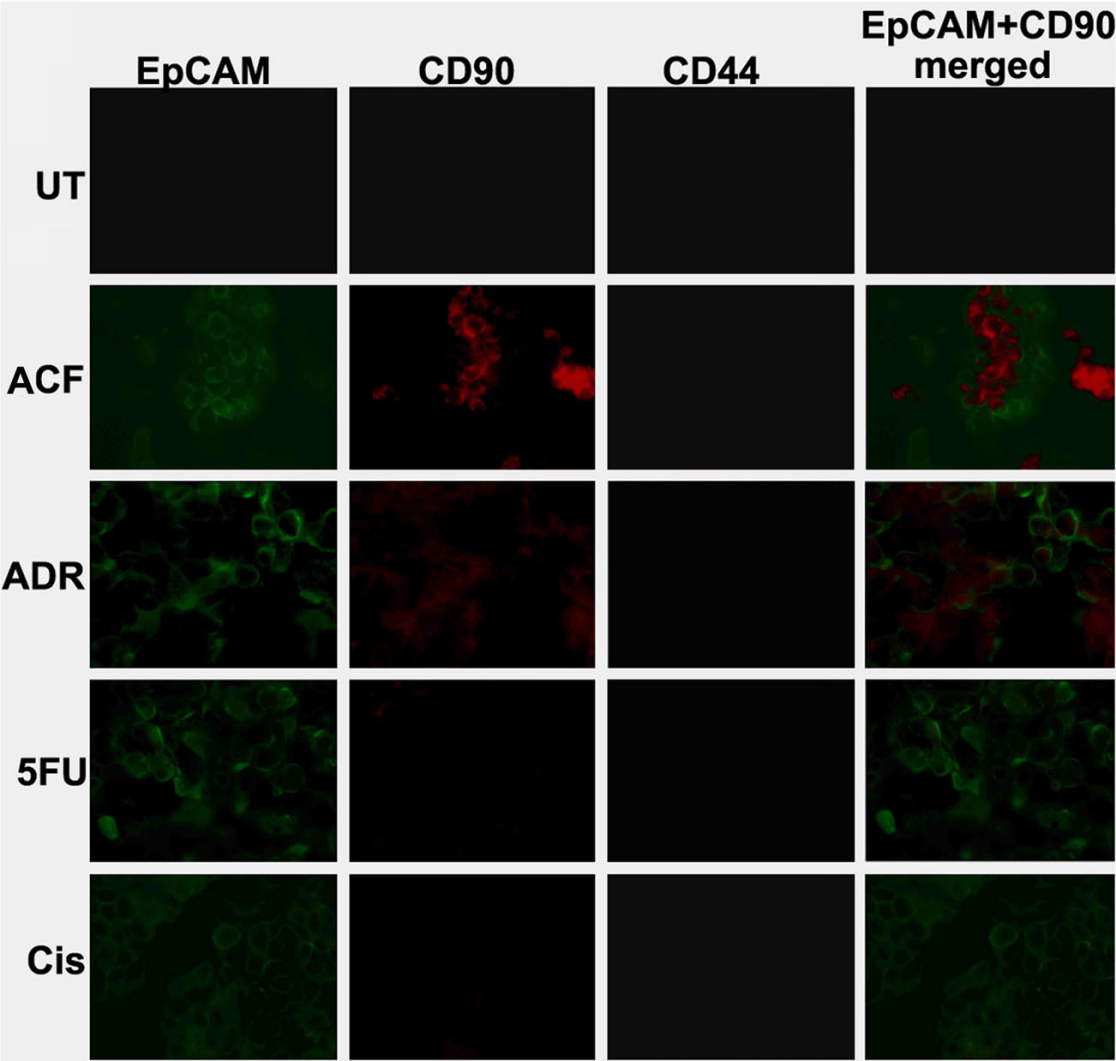
**EpCAM and CD90 expression in OE-19 spheres after drug treatment.** Immunocytochemical staining showing co-expression of EpCAM (green color) and CD90 (red color) in OE-19 spheres after treatment with the drug combination ACF for 48 h, revealing cancer stem cell-like identity. No CD44 staining was detected. UT: untreated; ADR: Adriamycin; 5FU: 5-fluorouracil; Cis: cisplatin

To assess the CSC-like nature of the surviving cells after ACF treatment, immunocytochemical staining was performed to detect EpCAM expression in conjunction with the expression of other known CSC-associated surface markers. Based on previous reports, CD44 and CD90 were selected for this purpose [32, 33]. After exposure of OE-19 cell spheres to ACF for again 48 h, we found that the surviving cells were positive for both CD90 and EpCAM staining, but negative for CD44 staining (Fig. 4). This result indicates that the remaining cells after ACF treatment could potentially be CSC-like cells playing an important role in drug resistance. Although the presence of CSC surface markers has been accepted as a main feature of CSCs, functional features such as the ability to form spheroids in vitro and/or to initiate tumor formation in vivo must be present to confirm the CSC identity.

### Tumor-initiating capacity of EpCAM positive cells after drug challenge

**Fig. 5:**
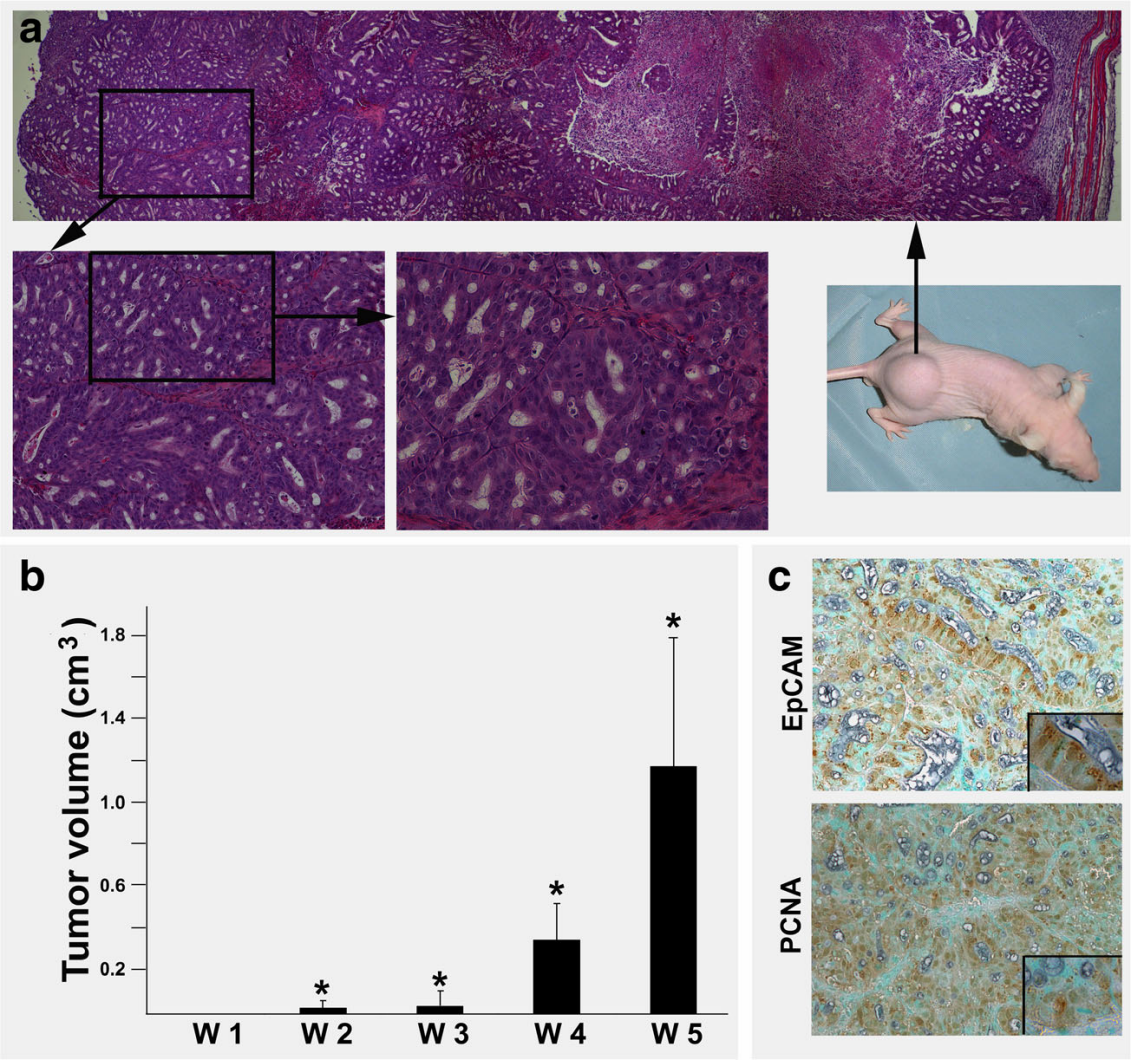
**Xenograft model for tumor formation of residual cells following drug treatment.** (**a**) Representative anatomy of tumors grown in nude mice inoculated with residual OE-19 cells following drug treatment (right lower: gross image). The histology of the tumor is shown in the low power field after H&E staining (upper, *: indicates necrotic zone). A detailed adenocarcinoma morphology is shown in higher power fields (left lower). (**b**) Tumor volume. W: week; *p < 0.05 versus W1. (**c**) Immunohistochemical staining showing EpCAM and PCNA (proliferation marker) expression in xenografted EAC tumor tissues

Next, we set out to test whether the residual cells after ACF treatment can initiate tumor growth. To this end, OE-19 cell spheres were exposed to ACF for 6, 12, 24, 48 and 72 h, after which ACF was removed and the remaining cells were cultured in spheroid medium (see materials and methods). We found that the cells that survived after ACF treatment for 6, 12 and 24 h again formed spheres within 3 days. We also found that it took about one week to form spheres for the cells that survived a 48 h ACF treatment. Since only few cells survived after 72 h of ACF treatment, we found that no spheres were formed by the remaining cells within 3 weeks (data not shown). To test the tumor-initiating capacity of OE-19 cells after ACF exposure, we selected the secondary (re-formed) OE-19 cell spheres after 48 h of ACF treatment for inoculation in nude mice. The mice were checked weekly for tumor formation. We found that these OE-19 cell spheres initiated aggressive tumor growth within 5 weeks. Tissues sections of the tumor bulk revealed histologic features characteristic of adenocarcinoma, whereas the aggressive growth pattern was represented by large tumor sizes and central necrotic zones (Fig. 5a, b). Subsequent immunohistochemical staining confirmed EpCAM expression, and the aggressive growth pattern was reflected by the presence of numerous PCNA-positive cells (Fig. 5c).

## Discussion

We observed increased EpCAM expression in primary malignant EAC tissues compared to that in adjacent benign esophageal epithelia. This increased expression was confirmed in vitro in EAC-derived cells compared to non-malignant Bar-T and HET 1-A cells. We found that the increase in EpCAM expression was associated with the occurrence of CSC features. The increased proportion of EpCAM-positive cells in OE-19 cell-derived spheres and in residual cells after drug treatment indicates that EpCAM may play a role in the acquisition of EAC chemoresistance. This is the first report showing that treatment of EAC cells with standard CTA, originally directed against ESCC cells [34], selects for a subset of cells, i.e. CSC-like cells, with a chemo-resistant and aggressive malignant potential. We found that CTA treatment not only led to increased EpCAM expression at early stages, but also to a selection of more aggressive tumor cells with a continued increased EpCAM expression, even post-treatment. This observation may have implications for what is now considered as standard of care for patients undergoing chemotherapy with respect to both treatment response and prognosis. Our data are concordant with those reported by Latifi et al. [18] for ovarian cancer, i.e., their data showed a similar increase in EpCAM expression in ovarian cancer cells following cisplatin treatment. They suggested that in these cells EpCAM may function to decrease E-cadherin expression, which is related to epithelial-mesenchymal transition (EMT) and CSC transformation [35–37]. It has been reported that EpCAM may undergo regulated intramembrane proteolysis (RIP), which results in shedding of the extracellular domain (EpEX) and release of the intracellular domain (EpICD) into the cytoplasm [38]. The EpICD may form a complex with β-catenin, which subsequently translocates to the nucleus. This, in turn, may lead to activation of canonical Wnt signaling [39], which contributes to the maintenance of stem cell pluripotency [40] and to the reprograming of somatic cells to pluripotency [41]. It has indeed been shown that EpCAM may induce a CSC-like gene signature in HBV-associated hepatocellular carcinomas [42]. Here, we observed co-expression of CSC markers, EpCAM and CD90, in OE-19 cells after ACF treatment. The exact mechanism underlying the acquisition of CSC features in EAC cells after CTA treatment requires further analysis, especially with respect to the role of the CSC-like cells in chemoresistance and tumor recurrence.

Given the relative abundance of EpCAM in epithelial carcinomas [10], it may well serve as a useful diagnostic/prognostic biomarker and as a suitable therapeutic target. Concordantly, Mitas et al. [17] already included EpCAM in their molecular EAC scoring system. Osta et al. [43] have reported that similar adeno-carcinomatous tumor cells (i.e., breast adenocarcinoma cells) may show a decreased malignant and metastatic potential following siRNA-mediated EpCAM silencing, as well as a reversal of E-cadherin derangements seen in EpCAM positive cells. A monoclonal antibody directed against EpCAM (catumaxomab) has been developed and included in a phase II/III trial for the treatment of malignant ascites as a peritoneal infusion with good results [44]. However, its applicability as infusional therapy in EAC remains to be seen. A potential therapeutic utility might be considered when a patient’s primary tumor is positive for EpCAM expression. The purpose of this line of therapy would be to target circulating CSCs, a population of cells with a high metastatic capacity [45].

In conclusion, we found that the expression of EpCAM is increased in primary EAC tissues and in vitro in EAC-derived cells. We also found that treatment of EAC-derived cells with standard chemotherapeutic agents may lead to an increased EpCAM expression, as well as to an increased malignant potential and an increased drug resistance. Further insight into the role of EpCAM expression in the acquisition of drug resistance may lead to the design of novel (targeted) EAC treatment strategies.
